# Flow-driven assembly of VWF fibres and webs in *in vitro* microvessels

**DOI:** 10.1038/ncomms8858

**Published:** 2015-07-30

**Authors:** Ying Zheng, Junmei Chen, José A. López

**Affiliations:** 1 Department of Bioengineering, University of Washington, Seattle, Washington 98109, USA; 2 Center of Cardiovascular Biology, Institute of Stem Cell and Regenerative Medicine, University of Washington, Seattle, Washington 98109, USA; 3 Bloodworks Northwest Research Institute, Seattle, Washington 98102, USA; 4 Departments of Medicine (Hematology), Biochemistry, and Mechanical Engineering, University of Washington, Seattle, Washington 98195, USA

## Abstract

Several systemic diseases, including thrombotic thrombocytopenic purpura, manifest much of their pathology through activation of endothelium and thrombotic occlusion of small blood vessels, often leading to multi-organ failure and death. Modelling these diseases is hampered by the complex three-dimensional architecture and flow patterns of the microvasculature. Here, we employ engineered microvessels of complex geometry to examine the pathological responses to endothelial activation. Our most striking finding is the capacity of endothelial-secreted von Willebrand factor (VWF) to assemble into thick bundles or complex meshes, depending on the vessel geometry and flow characteristics. Assembly is greatest in vessels of diameter ≤300 μm, with high shear stress or strong flow acceleration, and with sharp turns. VWF bundles and webs bind platelets, leukocytes and erythrocytes, obstructing blood flow and sometimes shearing passing erythrocytes. Our findings uncover the biophysical requirements for initiating microvascular thrombosis and suggest mechanisms for the onset and progression of microvascular diseases.

## Abstract (web summary)

3D microvessels with complex geometries and intact endothelium can be built *in vitro*. Using these engineered microvessels, here the authors show that the generation of the pathologic meshwork of the blood protein von Willebrand factor is affected by vessel architecture, flow and the proteolytic activity of ADAMTS13.

## Graphical abstract

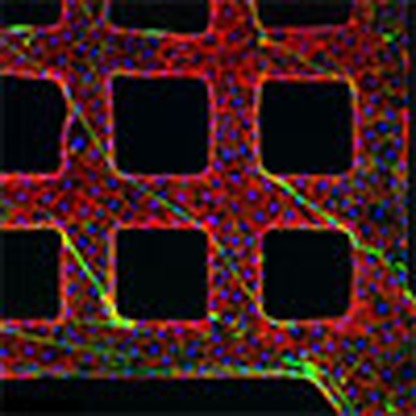

von Willebrand factor (VWF), a very large, multimeric, blood protein, has a pivotal role in initiating haemostasis and thrombosis and has emerged as an important risk factor and therapeutic target for many vascular diseases^1^,^2^,^3^,^4^. VWF is primarily secreted from the endothelium, either constitutively or in a regulated fashion from Weibel–Palade bodies after endothelial stimulation^5^,^6^. Much of the secreted VWF remains bound to the endothelial surface until it is proteolytically removed by the metalloprotease ADAMTS13 (ref. 7). Endothelium-attached VWF unfolds under fluid shear stress and flow acceleration, becoming more adhesive to bind platelets^8^, and more susceptible to ADAMTS13 proteolysis^7^. Failure to remove endothelium-bound VWF allows individual multimers to self-associate to form long strands that facilitate platelet adhesion and thrombus formation, which promotes microvascular occlusion in a group of life-threatening disorders that include thrombotic thrombocytopenic purpura (TTP)^2^, haemolytic uraemic syndrome^9^ and other vascular diseases^10^,^11^. In these pathologies, VWF multimers in plasma are often abnormally large and abundant, and, in TTP, terminal arterioles and capillaries become occluded by platelet- and VWF-rich thrombi^2^. However, the mechanisms of these diseases are not fully understood. In particular, it is not known why only the small vessels are affected, or whether platelets themselves are always necessary for the development of occlusive thrombi in TTP, which often worsens clinically even in the face of severe thrombocytopenia.

Fluid shear stress is an important regulator of VWF’s ability to bind platelets, as it unfolds the VWF molecule and renders it competent to bind platelets^12^. Nevertheless, how flow and vessel characteristics modify the structure and functions of VWF strands bound on the vessel walls have not been well studied, because it is difficult to directly image VWF strands with high resolution in small vessels *in vivo*.

Here, using a fully endothelialized system of *in vitro* microvessels that recapitulate the complex architectures and flow characteristics found *in vivo* (Fig. 1a,b), we examined the effects of haemodynamics and vessel geometry on the assembly of thin VWF strands into thicker strands or fibres and on their interactions with platelets and other blood cells. We found that the extent of strand formation and thickening depends on vessel architecture, flow and the proteolytic activity of ADAMTS13. As vessels become smaller, VWF strands become thicker and longer. Turns and bifurcations in vessels promoted VWF strand thickening, as did flow acceleration. In regions with complex flow, VWF formed three-dimensional (3D) web-like structures capable of blocking flow. Our study recapitulates the characteristics of thrombotic microangiopathies, and suggests that flow-driven assembly of VWF to thick and long fibres in small vessels has an essential role in the pathophysiology of these disorders.

## Results

### Geometry-mediated assembly of VWF strands and fibres

**Figure 1:**
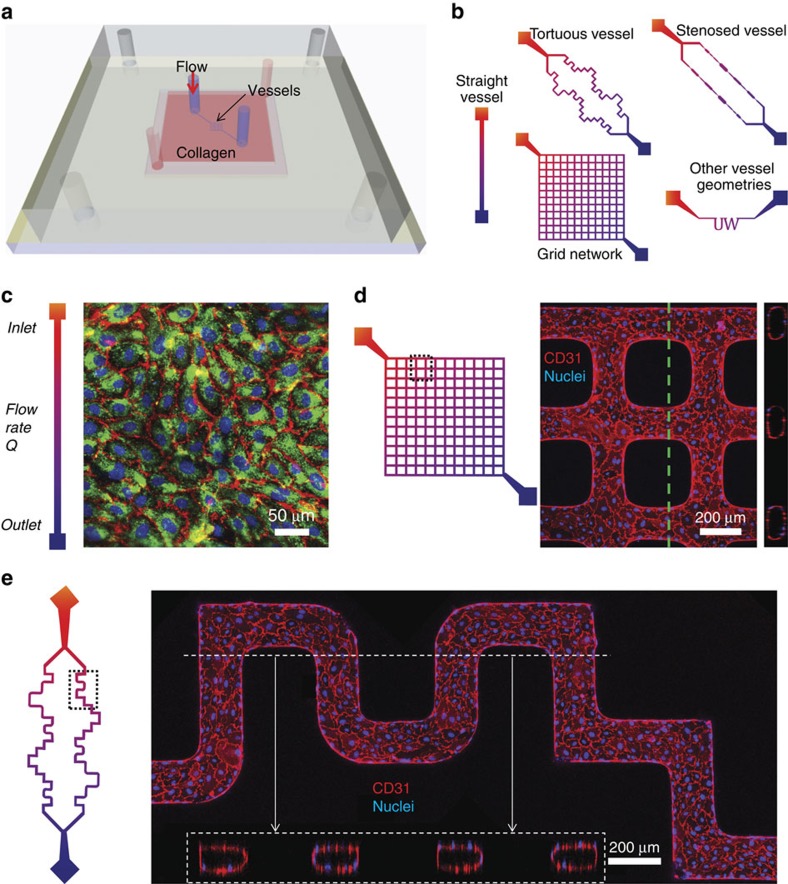
Microvessel system. (**a**) Schematic of the microvessel system in collagen gels. (**b**) Schematics of the different microvessel geometries: straight vessel, grid network, tortuous vessels, stenosed vessels and geometries using fonts or symbols. (**c**) Endothelial cells from a vessel that forms a large straight channel. Note the abundant VWF in Weibel–Palade bodies. Red: CD31, green: VWF and blue: nuclei. (**d**) z-projection of confocal sections of endothelialized grid microvessel networks and its cross-sectional view (right panel). (**e**) z-projection of confocal sections of endothelialized tortuous microvessels and cross-sectional views (bottom). Red: CD31 and blue: nuclei. Number of replicates for each type of vessel >10.

We engineered microvessel networks in type I collagen (7.5 mg ml^−1^) using lithographic processes that we described previously^13^ (Fig. 1a). We varied vessel diameters and used several microvessel geometries, including straight single-channel vessels, tortuous vessels with multiple turns, grid vessels with many junctions and bifurcations, stenosed vessels, and others with variable diameter and curvature (Fig. 1b). Human umbilical vein endothelial cells were seeded in the channels and cultured under gravity-driven flow for 1–2 weeks to achieve a confluent and continuous endothelium (Fig. 1c–e). The endothelium expressed CD31 at regions of cell–cell contact, and contained abundant granules rich in VWF, which were predominantly located in the cytoplasm in perinuclear regions (Fig. 1c).

**Figure 2:**
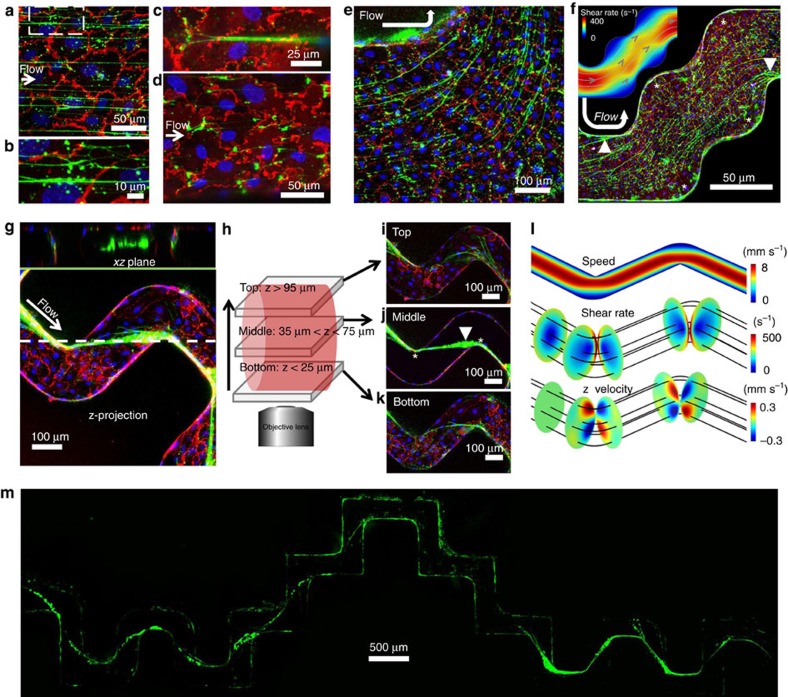
Assembly of VWF strands depends on vessel diameter and turns. (**a**,**b**) Secreted VWF from the activated endothelium formed strands along the direction of flow on a single straight vessel with a diameter of 500 μm (**b**. zoomed-in view of A, showing neighbouring parallel VWF fibres assembled to thicker strands). (**c**,**d**) Secreted VWF near the luminal wall of a vessel with diameter of 150 μm, (**c**) multiple strands from one or two cells converged to one thicker strand and (**d**) VWF strands follow the major direction of flow, but show some deviation due to surface irregularities. (**e**) VWF strands follow vessel turns, changing directions and remaining bound to the vessel wall in a 500-μm diameter vessel. (**f**) VWF strands remain bound to the vessel wall in a tortuous vessel with a diameter of 500 μm. Arrowheads: individual VWF strands self-associated into thicker strands in regions of high shear stress; asterisks: regions of low shear stress lack VWF strands; green: VWF, blue: nuclei. The upper left corner of the panel shows the COMSOL simulation of flow streamlines (white lines) and shear rate colour map at the cross-sectional plane with a distance to the bottom wall of 5% of the vessel diameter. (**g**) z-stack projection and cross-sectional view of confocal image of VWF transluminal bundles through microvessels of diameter <200 μm. (**h**) z-section projection of confocal images of **g** near the top wall (I: z >95 μm), vessel centre (J: 35 μm <z <75 μm) and the bottom wall (K: z <25 μm). Red: CD31, green: VWF and blue: nuclei. Asterisks: anchoring points of VWF fibres at the inner corners of vessel turns; arrowhead: self-association and thickening of VWF strands. (**l**) COMSOL simulation of flow through tortuous vessels showing the colour-coded flow speed at the centre longitudinal plane (top panel), shear rate at transverse cross-sections before and after the vessel turns (middle panel) and z-direction velocity at corresponding planes (bottom panel). (**m**) A continuous VWF strand of ∼5 cm in length (in green) extending through a torturous vessel along the shortest flow path. N >6.

When the vessels were stimulated with an endothelial secretagogue, the endothelium released VWF, of which a significant fraction remained bound to the endothelial surface and formed strands under flow. The pressure applied to each vessel ranged from 10 to 1,000 Pa between the vessel inlet and outlet during stimulation to generate an average wall shear stress of 5 dyn cm^−2^. In vessel regions that approximated straight tubes, individual VWF strands from adjacent streamlines assembled to form thicker fibres in the direction of flow (Fig. 2a,b), visible fibres ranging in thickness from 1 to 6 μm. This phenomenon occurred in all vessels with diameters between 100 and 1,000 μm (Fig. 2a–c). Near the vessel wall, most of the VWF strands followed the direction of bulk flow, but a few strands deviated from this pattern, likely because of small local flow disturbances induced by the surface irregularities on the endothelial monolayer (Fig. 2d). This effect was more prominent in vessels <200 μm in diameter. In larger vessels (≥500 μm) with one turn, the VWF strands followed the direction of flow and formed thicker strands near the inside corner of the turn (Fig. 2e). In larger vessels with multiple turns, the VWF strands (Fig. 2f) followed the streamlines and remained close to the vessel walls, within a distance of 20 μm from the vessel wall (∼5% of the vessel diameter). More and thicker VWF strands appeared at regions of high shear stress, some reaching a thickness of up to 20 μm at regions of peak shear stress (arrowheads, Fig. 2f). In regions where the shear stress was below 0.5 dyn cm^−2^, as in vessel concavities, no VWF strands were visible, the VWF remained globular (asterisks, Fig. 2f). A minimal flow shear stress appeared to be required in the microvessels, of ∼0.3 dyn cm^−2^ for VWF strand formation. Below this shear, VWF remained as punctate globules (Supplementary Fig. 1a–c).

In vessels under 200 μm in diameter containing multiple turns, which simulate tortuous vessels, the secreted VWF not only formed strands on the wall in the direction of flow, the strands also lifted away from the wall, and assembled into thick fibres towards the centre of the flow stream (Fig. 2g–k). Near the wall (Fig. 2h,i,k), a meshwork of 1–2-μm-thick VWF strands formed; these strands coalesced to form much thicker fibres and bundles at the centre of the flow stream (Fig. 2j), which anchored at the inside corners of the turns (asterisks, Fig. 2j). The thick VWF bundles occupied roughly 15% of the vessel cross-sections at the thickest region (arrowhead, Fig 2j, with a maximal cross-sectional area of ∼1,400 μm^2^), and spanned the vessel lumen in the shortest path between turns (Fig. 2g). Numerical simulation of flow in these regions predicted that peak flow would shift toward the inside corners where the shear stress is highest (Fig. 2l), consistent with previous studies^14^,^15^. Pairs of counter-rotating vortices developed in cross-sectional planes located immediately before and after the turns, which could serve as the driving forces for VWF accumulation in the centre stream of the tortuous vessel. The magnitude of the effect of these secondary flows on VWF self-association depended on parameters of vessel geometry, including the curvature, diameter and aspect ratio^16^. In the vessels with continuous turns and low aspect ratios (ratios of segment length between turns to diameter ≤4), the VWF strands formed continuous transluminal fibres throughout the entire vessel, reaching lengths of over 5 cm (Fig. 2m).

**Figure 3:**
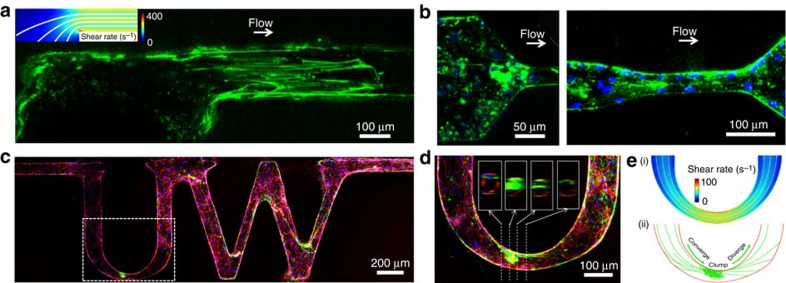
VWF structure is influenced by shear stress, flow acceleration and vessel curvature. In this figure, all of the vessels shown were activated with phorbol myristate acetate (PMA) before being perfused with buffer. (**a**) VWF deposition in a vessel segment one-fourth of the diameter of the segment that immediately precedes it. The calculated shear stress in the narrower segment was ∼3 dyn cm^−2^. (**b**) VWF clumps formed in stenosed microvessels of small internal diameter with flow acceleration. Green: VWF and blue: nuclei. (**c**,**d**) z-projection of confocal images of a stimulated ‘UW’ vessel. Red: CD31, green: VWF and blue: nuclei. (**d**) zoomed image of VWF structure near the narrowest region in (**c**), showing that the VWF clump blocks >50% of the vessel cross-sectional area. (**e**) (i) Wall shear rate in the U-shaped segment of the vessel, simulated with COMSOL. White lines: streamlines; colour: shear rate; and (ii) Schematic of VWF structures along the vessels when flow converges and diverges in the region of the curve. N >3 for each condition.

Flow acceleration also favored extension and thickening of VWF strands. For example, in a region where the vessel narrowed by 75% (from 600 to 150 μm diameter), the wall shear increased 64-fold. In spite of this huge increase in shear stress, it appears that flow acceleration at the entrance of the narrow region was more important for strand thickening and extension, as most of the VWF fibres were visible in this transition zone (Fig. 3a). In smaller vessels with a similar magnitude of narrowing (from 200 μm or 100 μm to 50 μm) VWF clumped rather than formed fibres in the region of acceleration (Fig. 3b). The accumulation of VWF at regions of vessel narrowing could be further enhanced by increasing curvature. For example, in the U-shaped vessel depicted in Fig. 3c,d, flow accelerated as it approached the region of maximum curvature (2.5 mm^−1^) and decelerated once past this region. VWF fibres converged at this region of maximum curvature, which was also the narrowest, and formed clumps that occluded almost 80% of the cross-sectional area (Fig. 3d). Downstream of this region, the VWF fibres diverged into thinner strands, some of which attached to the opposite wall of the vessel (Fig. 3d,e).

Thus, VWF self-association was influenced both by vessel diameter and curvature, and by shear stress and flow acceleration. VWF strands were thickest in smaller vessels with sharp turns, and at regions of narrowing.

### Flow-induced 3D VWF webs

**Figure 4:**
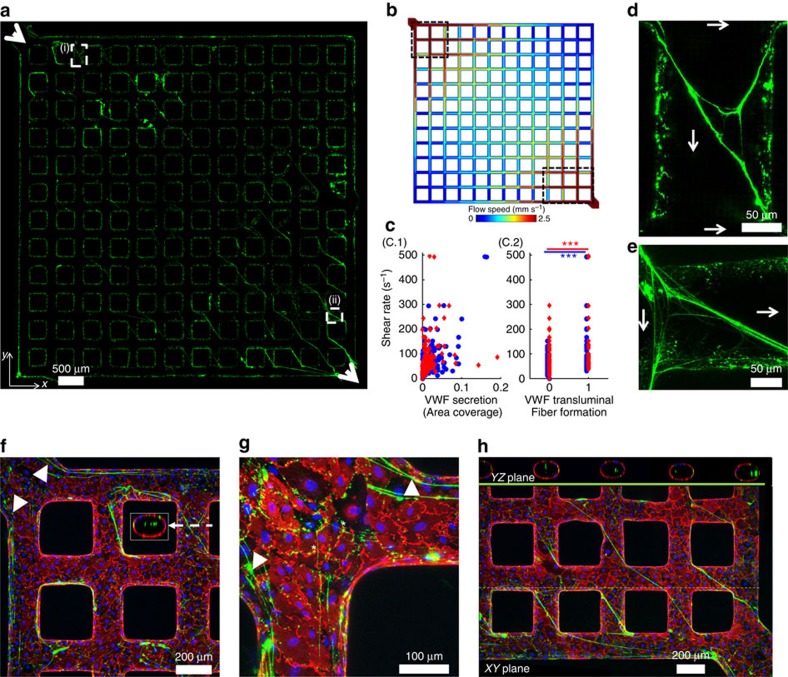
Flow-mediated formation of thick VWF fibres and meshes in grid vessels. (**a**) z-section projection of secreted VWF in a grid vessel network for −20 μm <z <20 μm (z=0 at the centre plane between the top and bottom walls of the vessel) showing that transluminal fibres formed predominantly near the inlet and outlet regions of the grid. Region (i) corresponds to panel **d** and (ii) corresponds to panel **e**. (**b**) COMSOL simulation of flow at the centre cross-sectional plane of the grid vessel. Colour map: flow speed. (**c**) Correlation between the shear rate and the amount of secreted VWF (C.1) or the presence of VWFtransluminal fibres (0: no VWF-transluminal fibres and 1: presence of VWF-transluminal fibres). Red: converging vessel branches (towards outlet) and blue: diverging vessel branches (near inlet). ***P<0.001 with paired *t*-test. (**d**,**e**) Zoomed-in view shows the flow-induced formation of (**d**) VWF strands entangled at the vessel junctions near the inlet and (**e**) a VWF web at the centre stream of a vessel branch near the outlet. (**f**–**h**) Confocal z-projection images of an activated grid vessel near the inlet (**f**, the dashed arrow points to a box that shows the *yz*-projected image at the dashed line), at the bifurcation of the inlet (**g**), and at the outlet (**h**, *xy*-plane projection in the bottom panel and *yz*-plane projection in the top panel). Arrowheads: merging of multiple VWF strands to thicker strands; asterisks: two VWF strands that originate near each other travelling in different directions at the bifurcation. Red: CD31, green: VWF and blue: nuclei. N >10.

Native vessel plexuses are highly branched as blood passes from arterioles to capillaries and to venules. In these structures, blood flow first diverges into a region of high total luminal cross-sectional area and decelerates, then converges and accelerates as it approaches the efferent vessel. To approximate this flow pattern, we used a grid vessel network with 13 × 13 vessel branches between one inlet and one outlet (Fig. 1d). When the endothelium in the grid was activated, secreted VWF formed strands and transluminal fibres in many vessel branches, particularly in the branches near the inlet and the outlet (Fig. 4a), where the flow rate and shear stress were both high (Fig. 4b). No transluminal fibres were observed at the branches near off-diagonal corners, where the wall shear stress was nearly 1/50 of the shear stress at the inlet. In the grid, flow first accelerated as it left the large inlet and entered the first two branches in the plexus. Thereafter, flow decelerated as branching increased (Fig. 4b). Flow speed increased again as the branches merged and flow approached the outlet. The number of transluminal fibres and the quantity of visible VWF were both proportional to the local wall shear stress: more VWF strands and transluminal fibres formed at high-shear regions near both the inlet and outlet (Fig. 4c, Supplementary Fig. 2a,b). A minimum shear rate of 50 s^−1^ was required for the formation of transluminal fibres and VWF strands along the vessel wall, consistent with the data shown in Fig. 2f. In high shear regions with multidirectional flow, the assembled fibres formed complex meshes that could potentially trap blood cells (Fig. 4d,e).

At the entrance to the grid (Fig. 4f,g and Supplementary Fig. 2a), where the vessel first bifurcated, VWF strands extended ∼2 mm downstream along the outer walls of the vessel and multiple VWF strands merged to form thick fibres (arrowheads in Fig. 4f,g). Near the crotch of the first bifurcation, VWF strands from single cells or multiple cells extended downstream, and split where the flow stream diverged (asterisks in Fig. 4g). Near the outlet, long VWF fibres extended continuously across several branch points, traversing the vessel lumen in the shortest path between branch corners and becoming progressively thicker as they approached the grid outlet (some strands were as thick as 40 μm at the outlet; Fig. 4h). The thickness of the transluminal fibres and the abundance of VWF in the webs corresponded with extensive depletion of VWF stores in Weibel–Palade bodies (compare Fig. 1c and Supplementary Fig. 2c, right panel).

### Effect of platelet adhesion on ADAMTS13 cleavage

We perfused platelets only, suspended in buffer, through a grid vessel after it was activated by phorbol myristate acetate (Supplementary Fig. 3). The platelets bound the preformed VWF strands and fibres (a process involving the platelet membrane glycoprotein (GP) Ib-IX-V complex^7^) producing mural and transluminal thrombi, which were larger in regions where the VWF was most abundant. The platelet–VWF strings/thrombi persisted throughout the 15 min of platelet perfusion, with minimal strand breakage.

**Figure 5:**
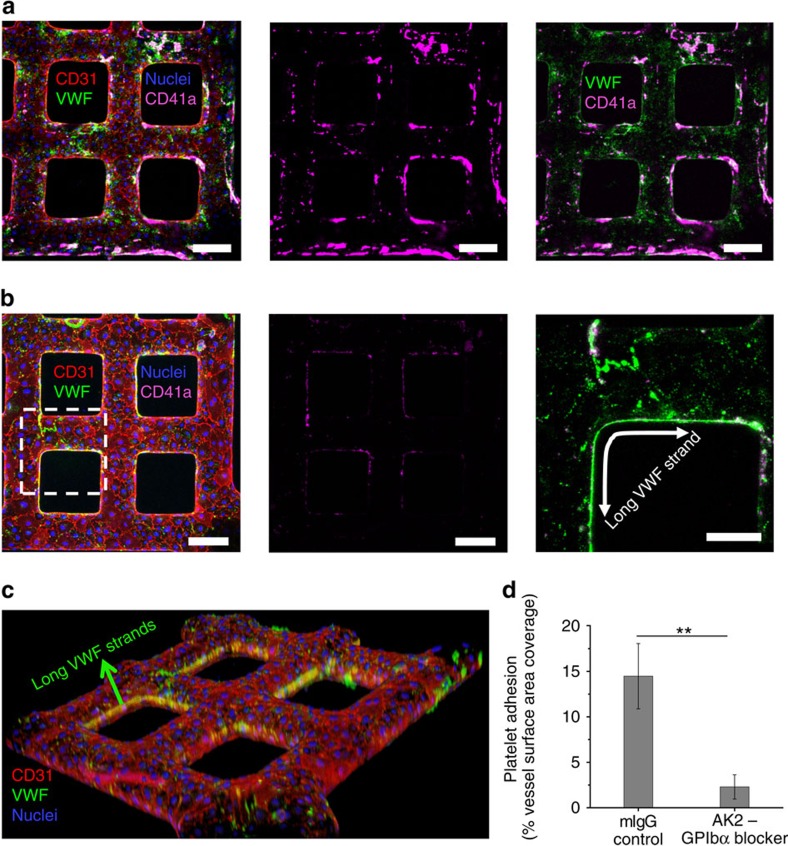
Effect of platelet adhesion on ADAMTS13 cleavage of VWF strands. (**a**,**b**) Confocal z-stack projection of activated grid vessels perfused with whole blood without (**a**) or with (**b**) the GPIbα blocker AK2: merged (left panel), platelets (middle panel) and platelets-VWF (right panel). Red: CD31, green: VWF, magenta: CD41a and blue: nuclei. Scale bar: 100 μm. (**c**) 3D reconstructed image of an activated grid vessel near the outlet after 15 min of perfusion of whole blood containing AK2. Red: CD31, green: VWF and blue: nuclei. (**d**) Vessel wall surface area covered by adherent platelets after 15 min of blood perfusion in the presence of either control mIgG or AK2. Error bar: s.d. **P<0.01 with student’s *t*-test. Three replicates were done. N >3.

VWF contains a single site for ADAMTS13 cleavage within each VWF subunit (in the A2 domain)^17^; cleavage of VWF by this enzyme produces smaller and less adhesive VWF multimers, thereby reducing platelet accumulation. When we perfused whole blood from normal donors (containing active ADAMTS13) instead of isolated platelets through the stimulated grid vessels, the number and size of platelet thrombi were greatly decreased (Fig. 5a and Supplementary Movie 1), and only a few VWF-platelet strings were apparent. Antibody inhibition of platelet GPIbα (the VWF-binding subunit of the GPIb-IX-V complex) almost completely prevented platelet adhesion to the VWF strands (Fig. 5b–d and Supplementary Movie 2). Many VWF strands appeared to have detached from the vessel wall and embolized as intact strands, and some of these were trapped at bifurcation crotches (Fig. 5b,c). It appears that in the absence of platelets, ADAMTS13 cleaves the VWF strands on the vessel wall more slowly. The attachment of platelets apparently creates a drag force that opens the VWF A2 domain and exposes the cleavage site for ADAMTS13, allowing the enzyme to degrade the VWF strands into smaller pieces, consistent with previous observations that ADAMTS13 preferentially acts on platelet-VWF complexes under fluid shear stress^18^. In addition, the binding of platelets to VWF strands prevents the strands from forming tightly packed fibres, which further facilitates ADAMTS13 cleavage. When platelets cannot bind to the strands this allows the strands to self-associate into thicker strands that take longer to cut, but when they are cleaved embolize as large pieces.

### Effect of ADAMTS13 on VWF strand structure

**Figure 6:**
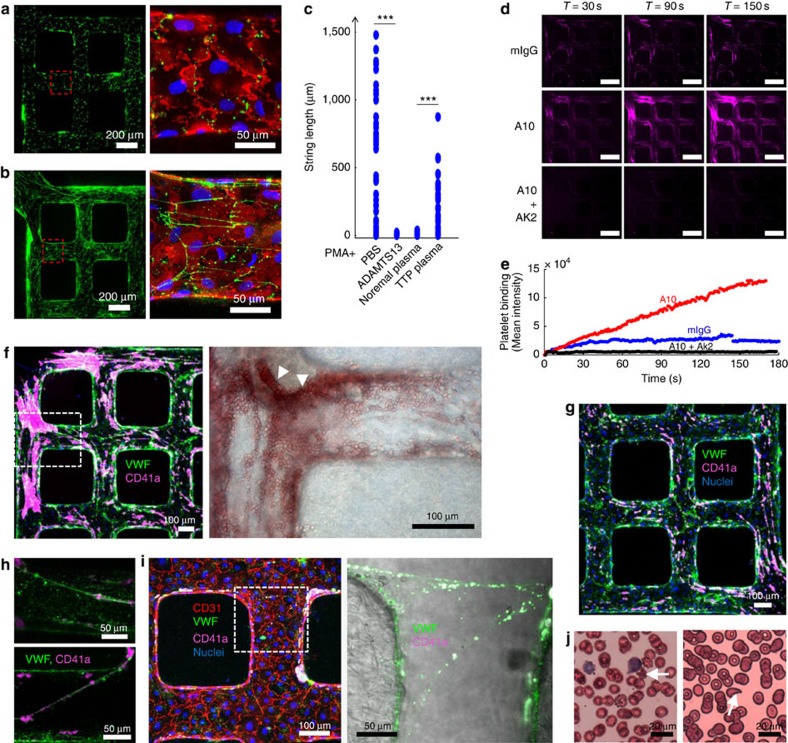
Effect of ADAMTS13 on VWF strand structure and thrombus formation. (**a**,**b**) PMA-stimulated microvessels in the presence of normal human plasma (**a**) or plasma from a TTP patient with 5% ADAMTS13 activity (**b**). The left panels in **a** and **b** show accumulated vessel wall VWF, and the right panels show zoomed-in views with vessel-wall staining also shown. Green: VWF, red: CD31 and blue: nuclei. (**c**) Comparison of length range of VWF strands from PMA-stimulated vessels perfused with: PBS, recombinant ADAMTS13, normal plasma or TTP plasma. ***P<0.001 with paired *t*-test. (**d**) Accumulation of fluorescent platelets on the vessel wall of stimulated vessels as a function of time with whole blood perfusion in the presence of control IgG (top panels), the ADAMTS13 inhibitory antibody A10 (middle panels), or the combination of A10 and the GPIbα inhibitory antibody AK2 (lower panels). Magenta: CD41a. Flow is from the top left to bottom right. Scale bar, 500 μm. (**e**) Quantification of adherent platelets on the vessel wall over time in (**d**). The size and number of thrombi are proportional to the mean fluorescence intensity of labelled platelets. (**f**,**g**) VWF-platelet strings/thrombi persists with significant platelet binding near the inlet (**f**) and less near the outlet (**g**) in the stimulated vessel after 30 min of whole blood perfusion with inhibited ADAMTS13. Partial blockage occurs with thrombi rich in platelets and VWF (**f**, left panel). Red blood cells accumulate at sites of platelet-VWF thrombi (**f**, right panel). Green: VWF, magenta, CD41a and blue: nuclei. (**h**) Zoomed view showing the structures of persistent VWF fibres bound platelets and red cells. Red: CD41a and green: VWF, white: colocalization of VWF and CD41a. (**i**) VWF persisted with minimal platelet binding in the stimulated vessel after 30 min of perfusion of blood with both ADAMTS13 and GPIb inhibited. Red: CD31, green: VWF and magenta: CD41a. (**j**) Blood film of blood treated with ADAMTS13 inhibitory antibody A10 and perfused through a stimulated microvessel showing red cell fragments and very few platelets. Arrows: red cell fragments. N >3 for each condition.

Deficiency of ADAMTS13 activity due to genetic mutation^19^,^20^ or neutralizing autoantibodies^21^,^22^ promotes microvascular thrombosis and development of TTP. We examined how ADAMTS13 deficiency affects the structure of VWF strands and webs. The microvessels were stimulated in the absence or presence of recombinant ADAMTS13 at a concentration of 1 μg ml^−1^ (a physiologic enzyme concentration, Fig. 4a and Supplementary Fig. 4), or in the presence of normal human plasma (Fig. 6a) or plasma from a TTP patient with <5% ADAMTS13 activity (Fig. 6b). Plasma with normal ADAMTS13 activity had the same effect as recombinant ADAMTS13, cleaving nearly all of the VWF strands during activation. Only small globules of VWF remained on the endothelial surface, likely representing the ‘stumps’ of VWF strands that remained at the sites of endothelial attachment (Fig. 6a and Supplementary Fig. 4c,d). In contrast, when TTP plasma was present during vessel stimulation, numerous VWF strands and complex webs accumulated on the vessel walls, and some of the strands spanned the vessel lumen (Fig. 6b). Vessels stimulated in the absence of ADAMTS13 had the longest VWF strands (Fig. 6c), with sizes ranging from globules of 1–2 μm to strands longer than the size of the field of view (>1.2 mm). The presence of ADAMTS13, either recombinant or in plasma, eliminated all of the long fibres on the wall or lumen; the maximum length of VWF strands left was ∼30 μm. In vessels activated in the presence of TTP plasma, the VWF strands were significantly longer than in the presence of normal plasma, spanning lengths between 1 and 800 μm. These strands were even thicker than those formed in the absence of plasma, sometimes even partially occluding the vessel lumens. The thickening of VWF fibres in the presence of TTP plasma was likely a result of association of fluid-phase VWF from the plasma with the immobilized endothelium-bound VWF strands^23^.

Next, we perfused stimulated vessels with blood containing inhibitory antibodies, blocking either ADAMTS13 alone or both ADAMTS13 and platelet GPIbα. In blood with control mouse IgG, the number of bound platelets plateaued after 1 min of perfusion (Fig. 6d,e). When the ADAMTS13 inhibitory antibody A10 (ref. 24) was added to the blood, platelet adhesion increased continuously over the 10 min that the vessel was monitored (Fig. 6d,e and Supplementary Movie 3), consistent with persistence of thick VWF strands (Fig. 6f,g). In addition to the thick strands, some VWF bundles appeared as loosely associated assemblies of several thin fibres connected to each other through bound platelets (Fig. 6h). Blood treated with both A10 and the GPIbα inhibitory antibody AK2 showed almost no platelet binding (Fig. 6d,e and Supplementary Movie 4), even though VWF strands were still present (Fig. 6i). In the vessels perfused with ADAMTS13-inhibited blood, flow resistance increased threefold at the 10 min time point compared with the control or double-blocking condition. This resistance was accompanied by a marked drop in the platelet count in the vessel effluent. Accompanying these changes, the platelet thrombi were much more prominent at the inlet of the grid (Fig. 6f) than at the outlet (Fig. 6g) despite the fact that the two regions had similar quantities of VWF fibres.

**Figure 7:**
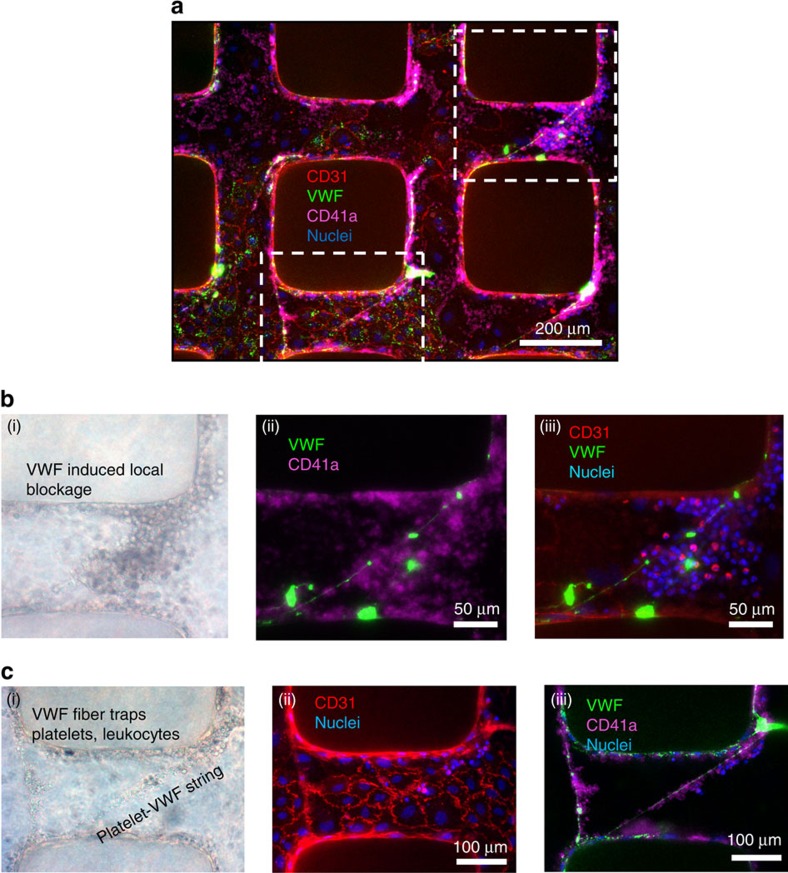
Effect of VWF antibody on platelet binding and thrombi formation. (**a**) VWF, when labelled with antibodies, resisted being cleaved by ADAMTS13 and trapped blood cells including platelets and leukocytes. (**b**,**c**) Zoomed images of microvessels in the dashed box in **a**, showing VWF fibres with bound platelet and leukocytes in the vessel lumen after 30 min of blood perfusion. (i) Bright-field images and (ii,iii) immunofluorescence projected z-stack images. In particular, (bii), (biii) and (cii) were z-stack projected images of planes from the centre plane to the wall, and (ciii) was z-stack projected image of centre planes of 20 μm thickness. Red: CD31, green: VWF, magenta: CD41a and blue: nuclei. N >3.

The platelet–VWF strings also bound erythrocytes (Fig. 6f). Curiously, it appeared that several layers of platelets accumulated at some sites before erythrocytes were able to attach (see arrowheads in Fig. 6f). There was also evidence of erythrocyte fragmentation (schistocytes) in the vessel effluent (Fig. 6j). In the presence of both A10 and AK2 (Fig. 6i), very few platelets or erythrocytes bound the VWF strands, suggesting that erythrocytes primarily adhere to immobilized platelets. Leukocytes also accumulated on the platelet/VWF strings when proteolysis of VWF was inhibited (Fig. 7 and Supplementary Movie 5).

## Discussion

Our studies have uncovered four important biophysical parameters that influence the development, thickness and length of VWF strands and webs in stimulated blood vessels: (1) vessel diameter, (2) vessel geometry (changes in curvature and diameter, bifurcations and convergences), (3) fluid shear stress and (4) flow acceleration. Vessel diameter influences two important phenomena: the ability of adherent platelets to occlude the vessel lumen, and the capacity of VWF strands to span the vessel lumen at turns. Within the limits of the vessel diameters we studied (40–1,000 μm), VWF strand thickness and length increased as vessel diameter decreased at equivalent shear stresses. In large vessels, VWF strands remained near the vessel wall and the influence of geometry was minimal. Therefore, bound platelets and other blood cells attached to the wall are more likely to exert local biological effects on the vessel wall rather than to occlude the lumen, an example being to initiate atherosclerotic lesions^25^. In smaller vessels with frequent turns, secreted VWF forms continuous strands that can span the vessel lumen and whose length is limited only by the length of the vessel. The longest we observed was ∼5 cm long. This is much longer than the longest VWF strands previously reported (several millimetres), which were formed on planar endothelial monolayers^7^. We do not mean to propose that VWF strands 5 cm in length commonly occur *in vivo* in pathologic situations, but rather to demonstrate the potential of VWF to form very long strands under the appropriate biophysical conditions. As opposed to the situation in larger vessels, platelet thrombi that form on smaller vessels are much more likely to occlude the vessel lumen, a process of pathophysiological importance in systemic diseases such as TTP^2^, sepsis^26^ and malaria^27^.

Another interesting observation we made relates to the pattern of platelet adhesion in grid vessels. In the absence of blood components, more VWF accumulates near the inlet and outlet of the grid, and when platelets are perfused through the vessel, the pattern of platelet adhesion reflects this pattern of VWF accumulation. However, the pattern of platelet adhesion was strikingly different when whole blood containing an ADAMTS13 inhibitor was perfused through the vessel. In this situation, platelets accumulated extensively near the inlet, partially obstructing flow and increasing the upstream pressure, while the platelet thrombi that formed near the outlet were much smaller, being insufficient even to coat all of the VWF strands (Fig. 6f,g). The reason for this difference is not readily apparent, but may have to do with the depletion of platelets near the inlet, leaving fewer to attach at the outlet. This phenomenon may have an *in vivo* correlate, as the hyaline thrombi in TTP^2^ are seen in arterioles and capillaries, but rarely, if ever, are seen in venules, despite the fact that these vessels have plentiful VWF.

This study highlights the mechanisms underlying the extremely low platelet counts observed in human TTP. The endothelium has a tremendous capacity to bind platelets. With a total surface area of ∼700 m^2^ in an adult human^28^ and abundant VWF stores, the activated endothelium would consume the entire quantity of platelets in the blood to cover this surface area with a monolayer of platelets, if even a fraction of the VWF was released at once and the mechanisms for its removal (ADAMTS13) were absent.

In summary, we have used a recently developed *in vitro* microvessel system to investigate the properties of VWF secreted from the vessel wall. This microvessel system is very useful, yet has a number of limitations in its ability to mimic the situation *in vivo*. These include, (a) the experiments described here have only one cellular component, endothelial cells. *In vivo*, vessels interact with a variety of cell types, including those of the organ in which the vessel resides, cells of the blood, and perivascular cells such as pericytes and smooth muscle cells; (b) although the vessels in our *in vitro* system can have very complex geometry, they are produced within only a single two-dimensional plane, whereas *in vivo* the vessels travel within 3D; (c) the system also differs from the *in vivo* situation in that the vessels are constantly perfused by blood that is circulating and continually being replenished in its content of hormones and small molecule mediators. Nevertheless, this system allows us to vary a number of parameters independently, such as geometry, diameter, flow and blood components, allowing us to uncover the physical parameters that drive the formation of hyperadhesive VWF strands and webs, and to provide tenable explanations for previously unexplained clinical phenomena. In addition, this system allows the use of entirely human components, making our findings more immediately relevant to human disease.

## Methods

### Patients and normal blood donors

Plasma was collected from a TTP patient with written informed consent approved by the Institutional Review Board of the University of Washington. Blood collections from healthy normal donors with written consent were under protocols approved by either the Institutional Review Board of the University of Washington or the Western Institutional Review Board.

### Microvessel fabrication and culture

Type I collagen was prepared from rat tails to a stock concentration of 15 mg ml^−1^ (ref. 29), which was further diluted and neutralized to 7.5 mg ml^−1^ on ice before microvessel fabrication. The microvessels were fabricated via soft lithography and injection moudling^13^, which includes four major steps: (1) defining a microstructured silicone stamp, (2) injecting collagen in housing devices (both microstructured and flat pieces) and allowing for gelation at 37 °C, (3) sealing the two collagen layers to form the enclosed fluidic structure and (4) seeding human umbilical vein endothelial cells (Lonza) through the microchannels between the inlet and outlet and allowing them to attach before long-term culture with perfusion of medium. The microvessels were cultured with growth medium (single quote kits from Lonza) comprising endothelial base media with 2% fetal bovine serum, 1% penicillin/streptomycin, epidermal growth factor, bovine brain extract with heparin, hydrocortisone, ascorbic acid, gentamicin and amphotericin B.

### Live imaging of VWF secretion and whole blood perfusion

In the studies of VWF secretion, the microvessels were activated with phorbol myristate acetate (50 ng ml^−1^ in serum free medium) for 40 min at a pressure drop between the inlet and the outlet varying between 10 and 1,000 Pa, depending on vessel geometry, to generate an average wall shear stress of ∼5 dyn cm^−2^. The vessels were then washed with PBS buffer before being perfused with buffer containing a FITC-conjugated VWF polyclonal antibody (1:100 dilution, Abcam). The accumulation of VWF in the microvessels was monitored with bright field and fluorescence imaging using an inverted microscope (Olympus IX81). After the live-imaging experiment, the microvessels were immediately washed with PBS then fixed and stained for an endothelial marker (CD31, 1:50 dilution, Abcam) for further imaging. VWF was quantified in the stimulated vessels using Matlab software, with three replicates at each condition. To visualize the VWF remaining inside the cells after stimulation, the cells were permeabilized with 0.5% Triton-X and restrained with VWF antibody and anti-CD31. In some experiments recombinant ADAMTS13 at concentration of 1 μg ml^−1^ in PBS was added during vessel activation.

In the studies using whole blood, blood was drawn from normal healthy donors into 3.8% (0.129 M) sodium citrate, centrifuged at 120*g* for 15 min at room temperature, and the platelet-rich plasma (PRP) was separated from the and red blood cells. The volume ratio of PRP to red blood cells and buffy coat was recorded and used for blood reconstitution before perfusion. Platelets in PRP were labelled with a monoclonal antibody to CD41a conjugated with Percp-cy5.5 (final concentration of 2.5 μg ml^−1^) for 30 min at room temperature. The platelet count from our normal donors was in the range of 150,000–400,000 μl^−1^. The labelled platelets in PRP were reconstituted with red blood cells and buffy coat at the original volume ratio before perfusion. To inhibit ADAMTS13, PRP was incubated with the ADAMTS13 inhibitory antibody A10 (final concentration: 100 μg ml^−1^) for 1 h, followed by the incubation with the antibody to CD41a (2.5 μg ml^−1^) for 30 min at room temperature before being reconstituted with the other blood cells. To inhibit GPIbα on platelets, PRP was incubated with the GPIbα-blocking antibody AK2 (final concentration: 60 μg ml^−1^) together with the antibody to CD41a for 30 min at room temperature before reconstitution. To inhibit both ADAMTS13 and GPIbα, PRP was first incubated with antibody A10 at room temperature for 1 h then incubated with AK2 and anti-CD41a for 30 min. The reconstituted whole blood was perfused through the microvessels that had first been washed with PBS to remove the residual serum from the culture medium. The flow was set so that the applied wall shear stress was between 10 and 30 dyn cm^−2^ and the blood was perfused for 15 min to 1 h. The vessels were monitored through bright field and fluorescence imaging using an inverted microscope (Olympus, IX81). The fluorescence arising from individual platelets and aggregates attached to the vessel wall was recorded with a digital camera (CoolSNAP) at 1–5 frame per second, and analysed using SlideBook software. Platelet adhesion was quantified using Matlab software. Two to four replicates were carried out for each experimental condition. At the end of blood perfusion, the microvessels was immediately washed with PBS, then fixed and stained for CD31 for further imaging.

In some experiments, isolated platelets were used, resuspended in Tyrode’s buffer (3 × 10^5^ platelets per μl). In these experiments, blood was anticoagulated with Acid Citrate Dextrose (ACD) (0.1 M sodium citrate, 0.11 M glucose and 0.08 M citric acid) and platelets were isolated from PRP after a second centrifugation at 1,200*g* for 15 min at room temperature, as described previously^7^.

### Immunofluorescence staining and analysis

At the designated time points, the vessels were washed with PBS and the secreted VWF was stained with a FITC-conjugated VWF antibody (1:100 dilution, Abcam) before the vessels were fixed *in situ* with 3.7% formaldehyde and washed three times with PBS. All reagents were delivered through the microchannels. For immunohistochemical staining of CD31 and intracellular VWF, the devices were first incubated for 30 min in a blocking solution consisting of 2% bovine serum albumin and 0.5% Triton-X to permeabilize cell membranes. The devices were then incubated overnight with a rabbit polyclonal antibody to CD31 (1:50 dilution, Abcam), washed, then incubated with a goat anti-rabbit antibody conjugated with Alexa fluor 568 and a FITC-conjugated VWF antibody (1:100 dilution, Abcam) for 2 h. The intact microvessels were visualized using a Nikon A1R confocal microscope and fluorescent images recorded. Image stacks were accumulated with a z-step between successive optical slices of ∼2 μm. Cross-sections, projections, and 3D reconstructions were generated from z-stacks of images using ImageJ software with orthogonal projection, z-projection and 3D viewer.

### Quantification of secreted vessel-wall VWF

In the grid vessel, the average wall shear rate was calculated for each of 312 vessel segments from the numerical simulation. From the immunofluorescence data, images of secreted VWF in grid microvessels were imported into ImageJ software to calculate the surface coverage of VWF on the vessel walls to obtain the amount of secreted VWF for each segment. This was expressed as per cent of the vessel surface covered by VWF. In addition, whether a vessel segment contained transluminal fibres was scored as 1 if one or more transluminal fibres were present, or zero if the segment contained no transluminal fibres. These data were then imported in Matlab software to plot the correlation between the shear rate and the secreted VWF or the presence of transluminal fibres.

### Statistical methods

Paired *t*-tests were used to compare group means for data presented in Figs 2, 3, 4. A *P* value <0.05 (*P<0.05, **P<0.01 and ***P<0.001) was considered significant for all tests. In the bar plots, the data is presented as mean±standard error from three samples for each experimental condition.

### Numerical simulation

The flow characteristics within 3D tubes and networks were simulated with COMSOL Multiphysics software, package ver. 4.0. The Navier–Stokes equation was used as pre-defined in COMSOL. The stationary solver for laminar flow was chosen for the Navier–Stokes equation. The fluid properties were defined as follows: viscosity of 1 × 10^−3^ Pa s and density of 1 × 10^3^ kg m^−3^ for water. The inlet boundary conditions are laminar flow with constant pressure of 10–1,000 Pa, and the outlet boundary conditions are laminar flow with zero pressure.

## Additional information

**How to cite this article:** Zheng, Y. *et al*. Flow-driven assembly of VWF fibres and webs in *in vitro* microvessels. *Nat. Commun.* 6:7858 doi: 10.1038/ncomms8858 (2015).

## Supplementary Material

Supplementary Information Supplementary Figures 1-4

Supplementary Movie 1 Citrated blood was perfused through a PMA-stimulated microvessel network. Platelets (in magenta) bound to VWF strands and formed platelet-VWF strings/thrombi. These strings/thrombi were constantly formed and removed during blood perfusion.

Supplementary Movie 2 Few platelets (in magenta) adhered to VWF strands in a PMAstimulated microvessel network when GPIbα was blocked with antibody AK2.

Supplementary Movie 3 When ADAMTS13 was inhibited by antibody A10, platelet (in magenta) adhesion on VWF strands, and platelet-VWF thrombus buildup, in a stimulated microvessel were much more rapid than in Supplementary Movie 1, in which ADAMTS13 activity was normal. Very few platelet-VWF strings/thrombi were removed and the vessel was occluded during the perfusion.

Supplementary Movie 4 When blood was perfused through a stimulated microvessel in the presence of inhibitory antibodies to both ADAMTS13 and GPIbα, VWF strands remained on the vessel wall but very few platelets adhered to the strands.

Supplementary Movie 5 VWF strands in a stimulated microvessel were first labeled with a polyclonal antibody (image not shown) before perfusion of blood with normal ADAMTS13 activity. Platelets (in magenta) adhered to VWF strands and formed platelet-VWF strings/thrombi. These strings/thrombi were stable and persisted throughout the time of blood
