# Supplementary material for: Flow-driven assembly of VWF fibres and webs in in vitro microvessels
Source: Nat Commun. 2015 Jul 30;6:7858. doi: 10.1038/ncomms8858 (PMC4522708; doi:10.1038/ncomms8858)
Supplement: Supplementary Information — Supplementary Figures 1-4 [file ncomms8858-s1.pdf]

Supplementary Figures:

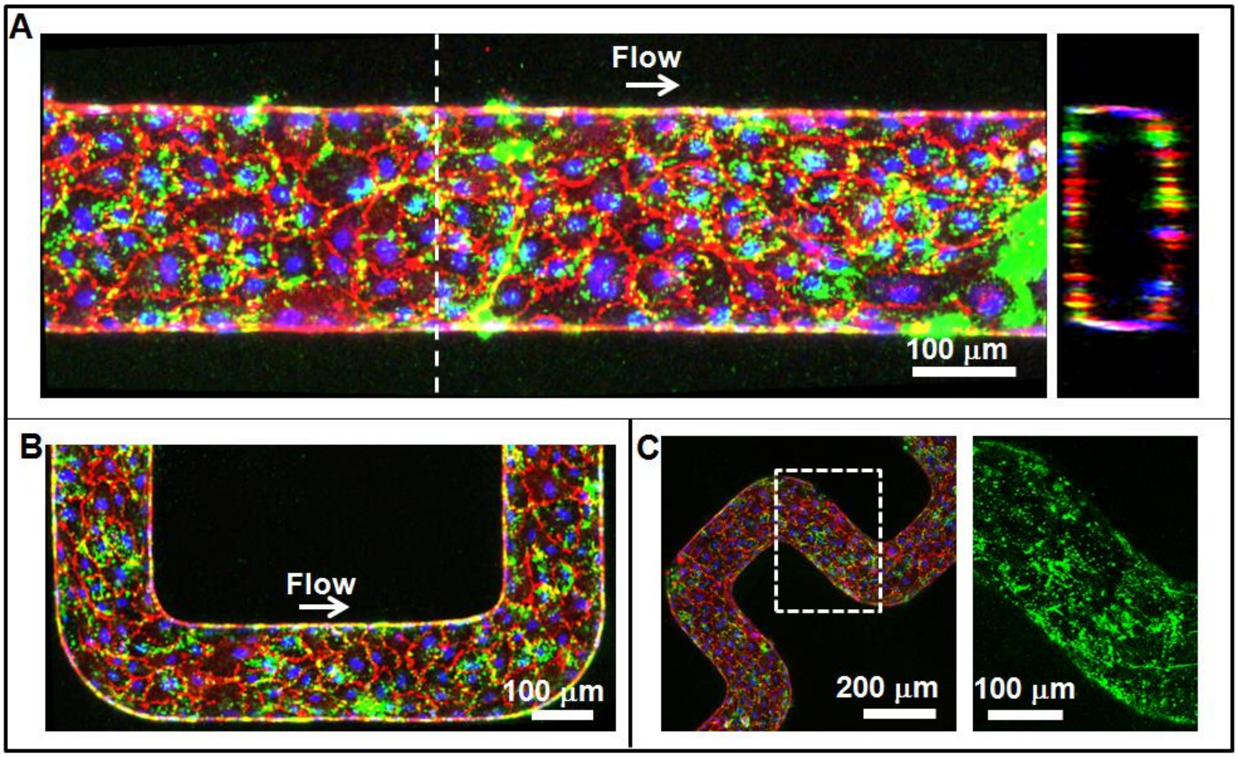

Supplementary Figure 1. *VWF structure is influenced by shear stress.* In this figure, all of the vessels shown were activated with PMA before being perfused with buffer. **A–B**. Two segments of the same vessel. In A, the segment is a straight tube with flow to generate a shear stress of 0.3 dynes cm^{-2}. B shows a curve segment downstream of the segment in A after the vessel has bifurcated into segments of smaller diameter. The shear stress in this curve segment is also 0.3 dynes cm^{-2}. At this shear stress, VWF was secreted but remained in clumps on the endothelial surface and did not form strands. **C.** One segment of a tortuous vessel stimulated at a averaged shear stress of approximately 0.3 dynes cm^{-2}. Note majority of endothelial-secreted VWF remained in clumps on the endothelial surface, several thin VWF strands formed near the vessel turns. Red: CD31, green: VWF, and blue: nuclei. Number of replicates N > 4.

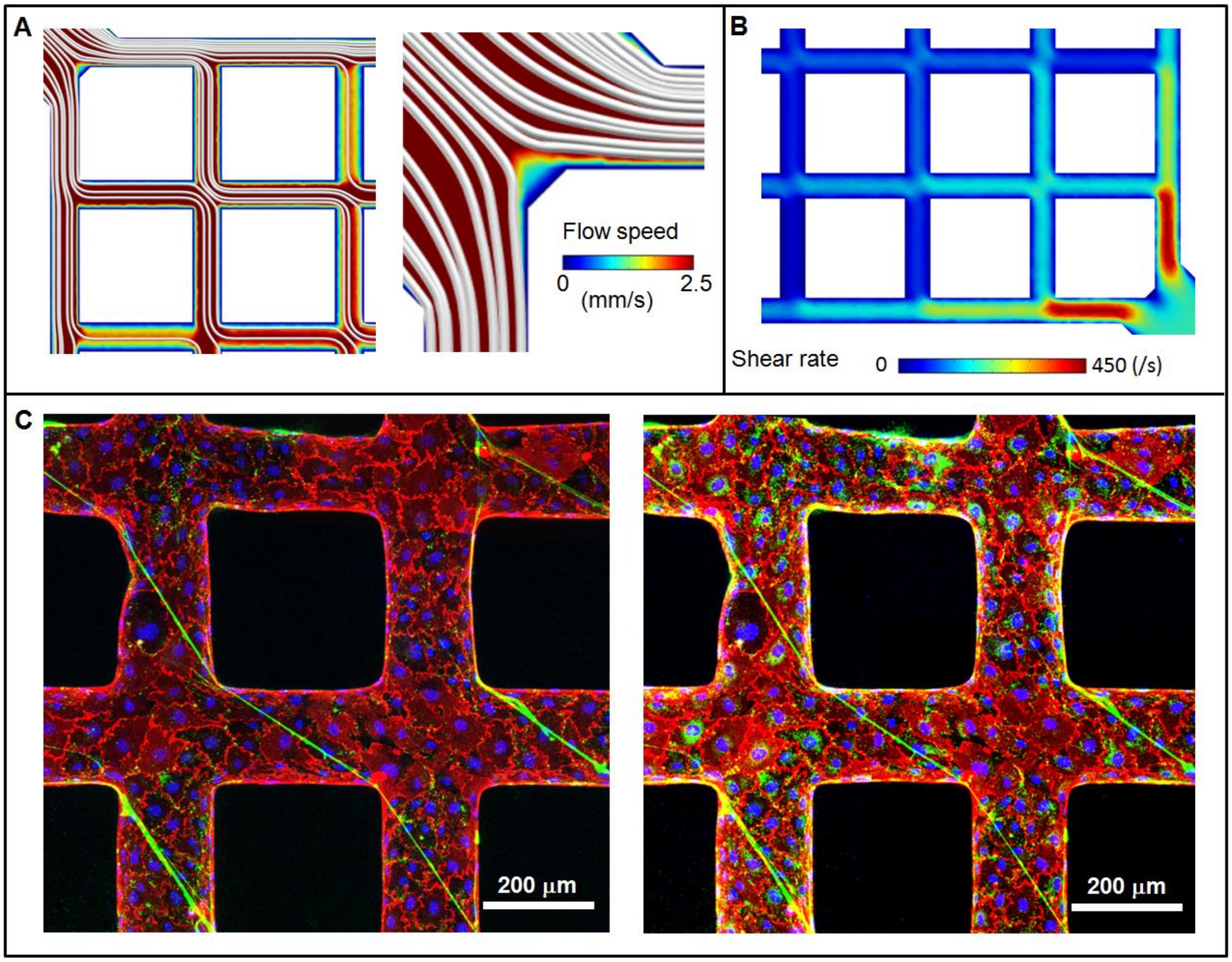

Supplementary Figure 2. *Flow velocity regulates formation of VWF strands in a grid network.* **A-B**. COMSOL simulation of 3D flow through a 13 x 13 grid network near the inlet (A) and the outlet (B). **C**. z-projection of confocal images of stimulated microvessels with the secreted VWF forming strands on the vessel wall and in the lumen (left panel) together with the remaining VWF within the endothelial cells (right panel). Red: CD31, green: VWF, and blue: nuclei. Number of replicates N > 6.

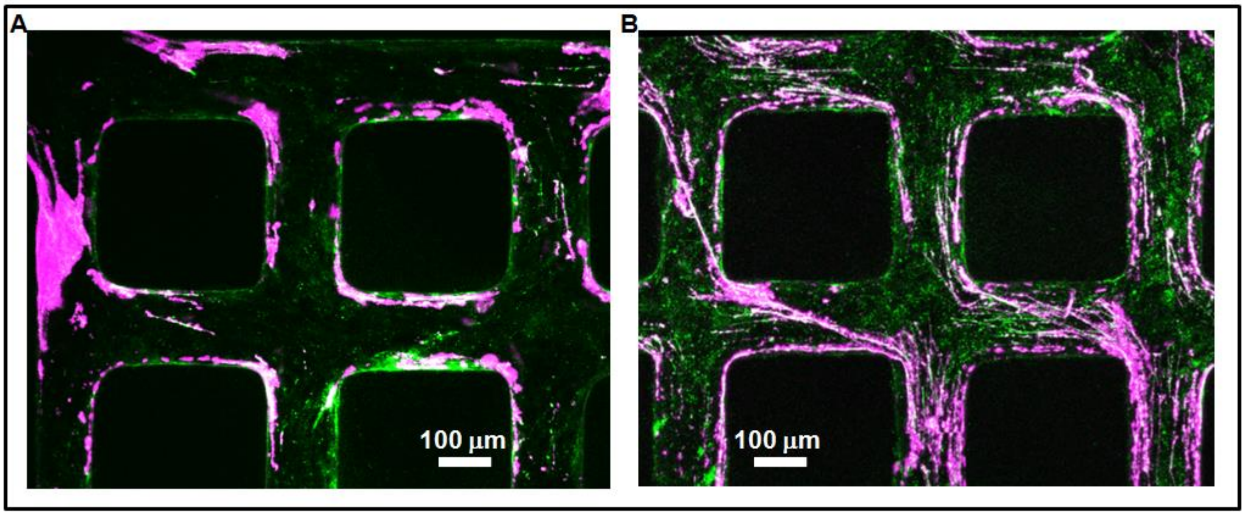

Supplementary Figure 3. *Platelet adhesion on complex VWF strands and fibers.* Isolated platelets perfused through stimulated microvessels of grid geometry for 15 minutes. **A**. Near the inlet, **B**. Near the outlet. Green: VWF, magenta: CD41a, white: colocalization of VWF and CD41a. Number of replicates N = 2.

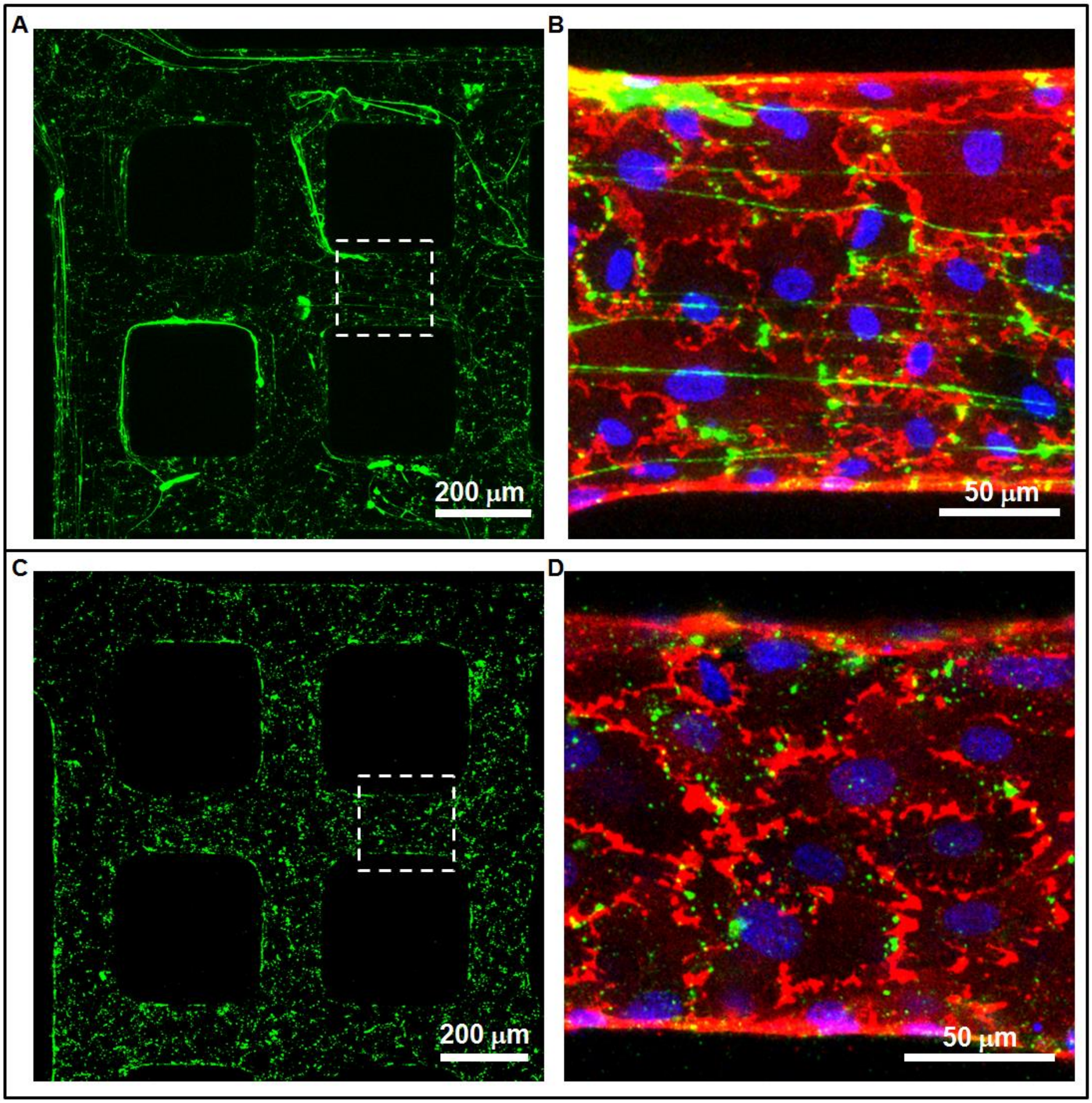

Supplementary Figure 4. *Effect of ADAMTS13 on VWF strand formation in microvessels*. **A-B**. z-projection of confocal images of microvessels stimulated in the absence of recombinant ADAMTS13, as shown in Fig. 2A (B: the zoomed images of microvessels in the dash box in A). **C-D**. z-projection of confocal images of microvessels stimulated in the presence of recombinant ADAMTS13 at 1µg/mL (D: the zoomed image of microvessels in dash box in C). Red: CD31, green: VWF, and blue: nuclei. Number of replicates N > 2.
